# Calcitriol Inhibits the Proliferation of Triple-Negative Breast Cancer Cells through a Mechanism Involving the Proinflammatory Cytokines IL-1*β* and TNF-*α*

**DOI:** 10.1155/2019/6384278

**Published:** 2019-04-10

**Authors:** Isela Martínez-Reza, Lorenza Díaz, David Barrera, Mariana Segovia-Mendoza, Sigifredo Pedraza-Sánchez, Giovanny Soca-Chafre, Fernando Larrea, Rocío García-Becerra

**Affiliations:** ^1^Programa de Investigación de Cáncer de Mama y Departamento de Biología Molecular y Biotecnología, Instituto de Investigaciones Biomédicas, Universidad Nacional Autónoma de México, 04510 Ciudad de México, Mexico; ^2^Departamento de Biología de la Reproducción Dr. Carlos Gual Castro, Instituto Nacional de Ciencias Médicas y Nutrición Salvador Zubirán, 14080 Ciudad de México, Mexico; ^3^Departamento de Inmunología, Instituto de Investigaciones Biomédicas, Universidad Nacional Autónoma de México, 04510 Ciudad de México, Mexico; ^4^Unidad de Bioquímica, Instituto Nacional de Ciencias Médicas y Nutrición Salvador Zubirán, 14080 Ciudad de México, Mexico; ^5^Laboratorio de Medicina Personalizada, Instituto Nacional de Cancerología, 14080 Ciudad de México, Mexico

## Abstract

Triple-negative breast cancer (TNBC) is one of the most aggressive tumors, with poor prognosis and high metastatic capacity. The aggressive behavior may involve inflammatory processes characterized by deregulation of molecules related to the immunological responses in which interleukin-1*β* (IL-1*β*) and tumor necrosis factor-*α* (TNF-*α*) are involved. It is known that calcitriol, the active vitamin D metabolite, modulates the synthesis of immunological mediators; however, its role in the regulation of IL-1*β* and TNF-*α* in TNBC has been scarcely studied. In the present study, we showed that TNBC cell lines SUM-229PE and HCC1806 expressed vitamin D, IL-1*β*, and TNF-*α* receptors. Moreover, calcitriol, its analogue EB1089, IL-1*β*, and TNF-*α* inhibited cell proliferation. In addition, we showed that synthesis of both IL-1*β* and TNF-*α* was stimulated by calcitriol and its analogue. Interestingly, the antiproliferative activity of calcitriol was significantly abrogated when the cells were treated with anti-IL-1*β* receptor 1 (IL-1R1) and anti-TNF-*α* receptor type 1 (TNFR1) antibodies. Furthermore, the combination of calcitriol with TNF-*α* resulted in a greater antiproliferative effect than either agent alone, in the two TNBC cell lines and an estrogen receptor-positive cell line. In summary, this study demonstrated that calcitriol exerted its antiproliferative effects in part by inducing the synthesis of IL-1*β* and TNF-*α* through IL-1R1 and TNFR1, respectively, in TNBC cells, highlighting immunomodulatory and antiproliferative functions of calcitriol in TNBC tumors.

## 1. Introduction

Triple-negative breast cancer (TNBC), which usually accounts for 5% to 20% of all types of human breast tumors, has high metastatic capacity, poor prognosis, and higher incidence in younger patients [1–3]. It is characterized by the lack of expression of estrogen receptor (ER), progesterone receptor (PR), and human epidermal growth factor receptor 2 (HER2) [4]. Given the absence of specific therapeutic molecular targets for this type of tumor, chemotherapy, radiotherapy, and mastectomy represent nowadays the mainstay for the treatment of affected individuals [5]. In recent years, the TNBC has been subclassified into 6 types based on its gene expression profile [6], with different behaviors among them, including response to treatment [7].

The aggressive behavior and poor prognosis of TNBC have been associated to inflammatory processes characterized by deregulation of molecules involved in the immune response [8]. In particular, interleukin-1*β* (IL-1*β*) and tumor necrosis factor-*α* (TNF-*α*) proinflammatory cytokines have an important role in the interaction between breast cancer cells and their microenvironment [9].

The cytokine IL-1*β* is a mediator of immune and inflammatory responses and exerts its biological effects by binding to two different membrane receptors, IL-1*β* receptor 1 (IL-1R1) that is a signaling receptor, leading to the activation of genes, and the IL-1*β* receptor 2 (IL-1R2) that lacks the intracellular domain and thus is incapable of signal transfer, which is why it is considered as dominant negative [10, 11]. Controversial functions have been attributed to this cytokine in breast cancer, including induction of migration and invasion or inhibition of cell proliferation [10, 12, 13].

TNF-*α* is another proinflammatory mediator with dual effects in breast cancer. Via its type 1 and type 2 receptors (TNFR1 and TNFR2), TNF-*α* may activate apoptosis, inhibit tumor growth, or promote tumor invasion, propagation, and aggressive behavior [14]. Depending on the cellular context, conditions, and microenvironment, TNFR1 activation may lead to the induction of apoptosis or necroptosis; however, the binding of TNF-*α* to TNFR2 most likely promotes cell proliferation [15–17].

On the other hand, low levels of calcitriol or its precursor calcidiol are associated with high risk of breast cancer incidence, progression, and aggressive behavior [18–21]. Calcitriol, via its nuclear vitamin D receptor (VDR), exerts antineoplastic properties by regulating several cell functions including growth, invasion, and cell apoptosis among others [22–24]. In addition, it has been demonstrated that vitamin D analogues with lower calcemic effects, such as EB1089, are also able to inhibit proliferation, stimulate differentiation, and induce apoptosis in breast cancer cells [25].

Calcitriol, as an immunomodulatory agent, has shown to differentially regulate the synthesis of both IL-1*β* and TNF-*α* cytokines in target tissues, including trophoblasts, leukemia cells, and human gingival fibroblasts [26–30]. In addition, CB1093, a calcitriol analogue, is known to increase TNF-*α*-induced cytotoxicity in ER-positive breast cancer cells [31]. However, little is known on the effects of calcitriol on IL-1*β* and TNF-*α* regulation in TNBC cells.

In addition, evidences from our laboratory and others have demonstrated that calcitriol enhanced the antiproliferative activity of antineoplastic agents, such as tyrosine kinase inhibitors, antiestrogens, radiotherapy, and chemotherapy [32–36].

The aim of the present study was to investigate the role of calcitriol on IL-1*β* and TNF-*α* gene and protein expression, including the effects of these cytokines on cell growth and their participation in the antiproliferative activity of calcitriol in TNBC cells.

## 2. Materials and Methods

### 2.1. Reagents

Cell culture media were purchased from Invitrogen (Thermo Fisher Scientific MA, USA). Fetal bovine serum (FBS) was from Hyclone Laboratories Inc. (Logan, UT, USA). Calcitriol (1*α*,25-dihidroxivitamina D_3_) was purchased from Sigma (St. Louis, MO, USA), seocalcitol (EB1089) was obtained from Tocris Bioscience (Bristol, United Kingdom), and IL-1*β* was purchased form R&D Systems (Minneapolis, USA). TNF-*α* was obtained from PeproTech (USA); TRIzol and the oligonucleotides for real-time polymerase chain reaction (qPCR) were from Invitrogen. The TaqMan Master reaction, probes, plates, and reverse transcription (RT) system were all purchased from Roche Diagnostics (Mannheim, Germany).

### 2.2. Cell Culture

The TNBC SUM-229PE (Asterand, San Francisco, CA) established cell line was cultured in Ham's F-12 medium supplemented with 5% heat-inactivated FBS, 10 mM HEPES, 1 *μ*g/ml hydrocortisone, 5 *μ*g/ml insulin, and 1% antibiotic-antifungal. The TNBC HCC1806 and ER-positive MCF7 cell lines (ATCC, Manassas, VA, USA) were cultured in RPMI 1640 medium with glutamine, supplemented with 5% inactivated FBS, 10 mM HEPES, 1 mM sodium pyruvate, and 1% antibiotic-antifungal. Cell cultures were kept in a humidified atmosphere with 5% CO_2_ at 37°C.

### 2.3. Western Blots

Cell protein homogenates (25 *μ*g) were separated by electrophoresis in 12% polyacrylamide gels, transferred to nitrocellulose membranes, and blocked overnight with 5% nonfat dry milk. The membranes were washed and incubated in the presence of the following monoclonal antibodies: anti-IL-1R1, anti-IL-1R2, anti-TNFR1, anti-TNFR2, anti-VDR (sc-393998, sc-376247, sc-8436, sc-393614, and sc-13133, respectively; Santa Cruz Biotechnology, CA, USA), and anti-glyceraldehyde 3-phosphate dehydrogenase (GAPDH, MAB374, Millipore, Milford, MA, USA) overnight at 4°C. Membranes were incubated in the presence of secondary antibody conjugated with horseradish peroxidase (sc-2031, Santa Cruz Biotechnology) for 2 hours at room temperature. The immunoblots were visualized by chemiluminescence using ECL Plus (Amersham Pharmacia, UK).

### 2.4. Proliferation Assay

Breast cancer cell lines were seeded in 96-well culture plates at a density of 1000-1200 cells/well depending on the cell line by triplicate. Then, the cells were treated in the absence or presence of different concentrations of calcitriol, EB1089 (0.01-100 nM), IL-1*β*, and TNF-*α* (0.05-100 ng/ml) or the combination of calcitriol with TNF-*α*. In addition, the cells were incubated in the presence of anti-IL-1R1 and anti-TNFR1 alone or in combination with calcitriol or the cytokines during 6 days at 37°C, 95% air, and 5% CO_2_ in a humid environment. After incubation, cell proliferation was determined using the colorimetric XTT Assay Kit (Roche), according to the manufacturer's instructions. Absorbance at 492 nm was measured in a microplate reader (BioTek, Winooski, VT, USA).

### 2.5. qPCR Analysis

To study the effect of calcitriol and its analogue EB1089 in the regulation of IL-1*β* and TNF-*α* mRNA, cell lines were cultured in the absence and presence of different concentrations of these compounds for 24 hours. After treatment, the cells were harvested and the RNA was extracted with TRIzol reagent. For the cDNA synthesis, a commercial kit was used (Transcriptor First-Strand cDNA Synthesis, Roche). For gene amplification, a set of specific probes and oligonucleotides for each gene was used (Table 1). The results were normalized against the constitutive gene GAPDH. Real-time PCR was carried out using the LightCycler 480 from Roche according to the following protocol: activation of Taq DNA polymerase and DNA denaturation at 95°C for 10 minutes, proceeded by 45 amplification cycles consisting of 10 s at 95°C, 30 s at 60°C, and 1 s at 72°C.

### 2.6. TCGA Data Analyses

A search in the Human Protein Atlas database (http://www.proteinatlas.org) was performed with the expression levels by RNAseq of IL-1*β* and TNF-*α* in Fragments Per Kilobase Million (FPKM) for 1075 patients with breast cancer from The Cancer Genome Atlas (TCGA) database. The optimal cutoff for IL-1*β* and TNF-*α* was evaluated with the X-tile and Cutoff Finder software [37, 38] for overall survival (OS). Survival analysis was evaluated through the Kaplan-Meier plot and the log-rank test in the SPSS software (SPSS Inc., Chicago, IL, USA). A *P* value < 0.05 was considered statistically significant.

### 2.7. Cytokine Measurements

The cell lines were cultured in the absence and presence of different concentrations of calcitriol and its analogue during 3 and 72 hours to IL-1*β* and TNF-*α*. The quantification of IL-1*β* and TNF-*α* concentrations in culture media was determined in triplicate by enzyme-linked immunosorbent assay (R&D Systems ELISA Kits) according to the manufacturer's protocol. The absorbance was quantified at a wavelength of 492 nm in a Multiskan MS photometer type 352 (Labsystems, Helsinki, Finland).

### 2.8. Statistical Analyses

Data are expressed as the mean ± standard deviation (SD). Statistical analyses were determined by one-way ANOVA followed by the Holm-Sidak method, using a specialized software package (SigmaStat, Jandel Scientific). Differences were considered statically significant at *P* < 0.05.

## 3. Results

### 3.1. Expression of VDR and Cytokine Receptors in TNBC Cell Lines

**Figure 1:**
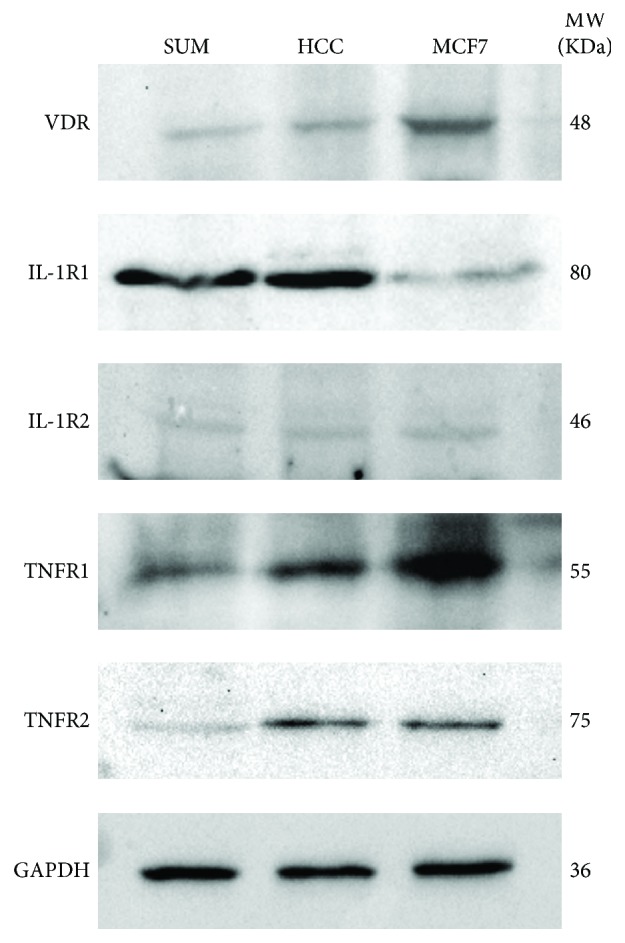
Expression pattern of VD, IL-1*β*, and TNF-*α* receptors in TNBC established cell lines. Cell lysates from untreated SUM-229PE (SUM), HCC1806 (HCC), or MCF7 were separated by SDS-PAGE. Thereafter, proteins were transferred to nitrocellulose membranes and incubated in the presence of specific antibodies. GAPDH was used as loading control. A representative image of a Western blot from two independent experiments is shown. VDR: vitamin D receptor; IL-1R: IL-1*β* receptor; TNFR: TNF-*α* receptor.

The basal protein expression of the VDR (48 kDa), IL-1R1 (80 kDa), IL-1R2 (46 kDa), TNFR1 (55 kDa), and TNFR2 (75 kDa) was studied by Western blots in TNBC cell lines. MCF7 cells were included as positive controls. As depicted in Figure 1, all cell lines studied showed the presence of all receptors tested, suggesting that TNBC cells are able to respond to calcitriol, IL-1*β*, and TNF-*α*. Of note, TNBC cells had higher IL-1R1 and lower VDR protein expression when compared to ER-positive cells. Between TNBC cells studied, SUM-229PE showed lower TNFR2 protein expression than HCC1806 cells.

### 3.2. Effects of Calcitriol and Cytokines on Cell Proliferation

**Figure 2:**
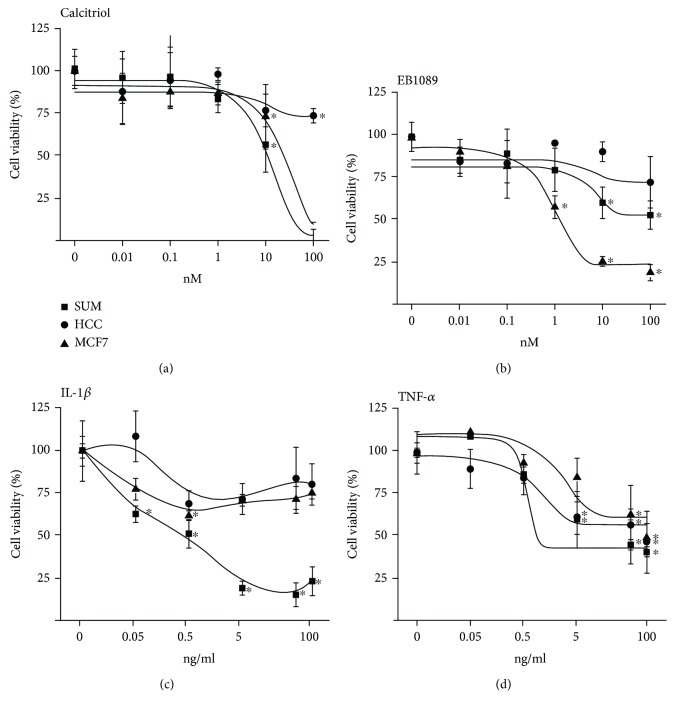
Calcitriol, EB1089, IL-1*β*, and TNF-*α* effect on breast cancer cell proliferation. SUM-229PE (■), HCC1806 (●), and MCF7 (▲) cells were cultured in the absence (0) or presence of different concentrations of calcitriol (a), EB1089 (b), IL-1*β* (c), or TNF-*α* (d) during 6 days. Then, cell proliferation was evaluated by the XTT method. The results represent the average of 3 experiments, each in triplicate ± SD. ^∗^ *P* < 0.05 vs. control. Nontreated cells were considered as 100% of cell proliferation. Ethanol was used as vehicle of calcitriol and EB1089 and phosphate-buffered saline solution (PBS) for cytokines.

The effects of different concentrations of cytokines and VDR agonists on breast cancer cell proliferation were evaluated using the XTT method. The results showed that the sensitivity of the cells to the compounds varied among the cell lines. As shown in Figure 2, calcitriol and its analogue EB1089 significantly inhibited the proliferation of SUM-229PE and MCF7 cells. Regarding cytokines, IL-1*β* significantly diminished the growth of SUM-229PE cells. In contrast, TNF-*α* did have inhibitory effects in the proliferation of all the three cell lines tested. Neither EB1089 nor IL-*β* had any effect on HCC1806 cell growth, while calcitriol significantly inhibited cell proliferation only at 100 nM.

**Figure 3:**
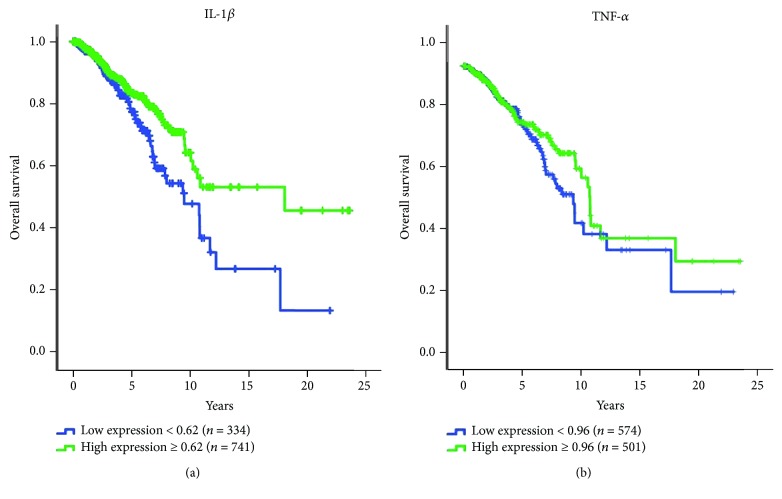
IL-1*β* and TNF-*α* expression in breast cancer patients. Kaplan-Meier plots showing the overall survival analysis of breast cancer patients according to IL-1*β* (a) and TNF-*α* (b) expression levels from TCGA databases.

Considering IL-1*β* and TNF-*α* effects in the proliferation of breast cancer cell lines, we decided to investigate the relation between the cytokine mRNA levels and survival of breast cancer patients using TCGA data retrieved from the Human Protein Atlas database. The results demonstrated that patients with high mRNA expression levels of IL-1*β* had a better prognosis than those with low levels. Patients with high IL-1*β* had a median overall survival (OS) of 18.0 years vs. 9.4 years for the rest (*P* = 0.007). Those cases with high TNF-*α* presented a median OS of 10.8 vs. 9.4 years (*P* = 0.249) though not statistically significant, there was a trend towards better prognosis. The optimal cutoff points were 0.62 and 0.96 FPKM for IL-1*β* and TNF-*α*, respectively (Figure 3).

### 3.3. Calcitriol Induced IL-1*β* and TNF-*α* Gene Expression and Secretion in TNBC Cells

**Figure 4:**
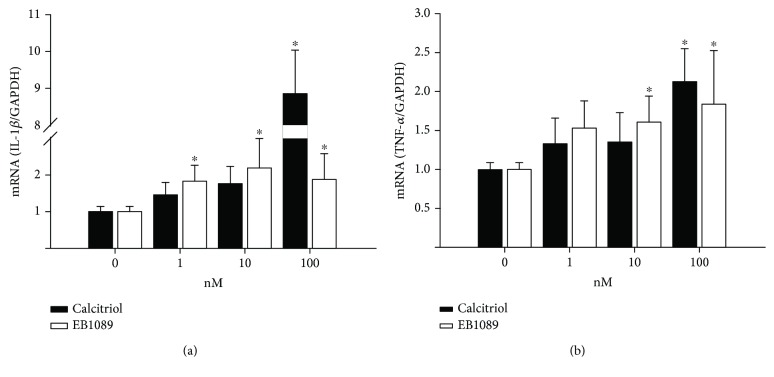
Calcitriol and its analogue increased IL-1*β* and TNF-*α* gene expression in SUM-229PE cells. Cells were cultured in the absence or presence of different concentrations of calcitriol (black bars) or EB1089 (white bars) for 24 hours. (a) IL-1*β* and (b) TNF-*α* gene expression was assessed by qPCR. The results represent the average of at least 3 experiments in triplicate ± SD. ^∗^ *P* < 0.05 vs. control (0). For data normalization, gene expression in cells without treatment was considered as one.

There is substantial evidence that calcitriol regulates the production of IL-1*β* and TNF-*α* in different tissues [26–28, 30]. Therefore, we decided to evaluate the effects of calcitriol and its analogue on the production of these cytokines in breast cancer cells.  Figure 4 shows that both calcitriol and EB1089 stimulated IL-1*β* and TNF-*α* gene expression in SUM-229PE cells. IL-1*β* mRNA expression levels significantly increased at a concentration of 100 nM of calcitriol and at all concentrations of EB1089 used (Figure 4(a)). A significant increase of TNF-*α* gene expression was observed at a concentration of 100 nM of calcitriol and at 10 nM in the case of EB1089 (Figure 4(b)). Regarding HCC1809 cells, calcitriol treatment significantly increased TNF-*α* mRNA levels only at 100 nM (Supplementary Figure S1b), while EB1089 induced IL-1*β* gene expression in MCF7 at all concentrations tested (Supplementary Figure S1c). Neither calcitriol nor its analogue significantly modified IL-1*β* gene expression in HCC1809 (Supplementary Figure S1a) or TNF-*α* in MCF7 cells (Supplementary Figures S1a and S1d, respectively).

Cytokine's secretion was also studied. As shown in Table 2, calcitriol at concentrations of 10 and 100 nM significantly increased TNF-*α* and IL-1*β* secretion, respectively, whereas IL-1*β* and TNF-*α* levels were significantly augmented by EB1089 at all concentrations examined in SUM-229PE cells. Regarding HCC1806 cells, the secretion of IL-1*β* was significantly increased only with EB1089 (1-100 nM). Neither calcitriol nor EB1089 modified IL-1*β* or TNF-*α* levels in MCF7 cells. These results demonstrated that calcitriol and its analogue have the capacity to modulate IL-1*β* and TNF-*α* response in vitro preferably in SUM-229PE cells; therefore, we chose this cell line to investigate the next objective of this study.

### 3.4. The Antiproliferative Effects of Calcitriol Were Reversed by Blocking IL-1R1 and TNFR1

**Figure 5:**
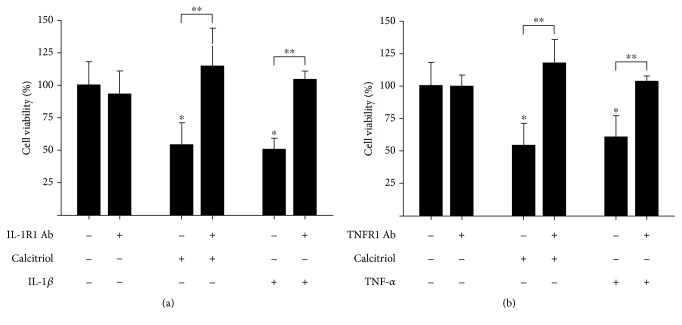
Calcitriol inhibited cell proliferation by inducing endogenous production of IL-1*β* and TNF-*α*. SUM-229PE cells were treated with calcitriol (10 nM), IL-1*β* (0.5 ng/ml), TNF-*α* (5 ng/ml), anti-IL-1R1 (5 ng/ml), and anti-TNFR1 (2 ng/ml), individually or combined as indicated. Cell proliferation was evaluated after 6 days of treatment using the XTT method. Vehicle-treated cells were considered as 100% of cell proliferation. The results represent the average of 3 experiments, each in triplicate ± SD. ^∗^ *P* < 0.05 vs. vehicle-treated cells (-). ^∗∗^ *P* < 0.05 calcitriol or cytokine alone vs. combined treatments.

In order to determine if calcitriol antiproliferative effects could be mediated through endogenous IL-1*β* and TNF-*α* synthesis, we performed proliferation assays in the presence of exogenous calcitriol, IL-1*β*, and TNF-*α*, with or without antibodies against IL-1*β*, TNF-*α*, or anti-cytokine receptor antibodies. As expected, calcitriol, IL-1*β*, and TNF-*α* caused a significant decrease in cell growth. Interestingly, the inhibitory effect of these compounds was significantly reversed when cells were treated with the combinations of calcitriol or IL-1*β* in the presence of anti-IL-1R1 (Figure 5(a)) and the combinations of calcitriol or TNF-*α* with anti-TNFR1 (Figure 5(b)). The anti-IL-1*β*, anti-IL-1R2, anti-TNF-*α*, and anti-TNFR2 had no effect on cell proliferation (data not shown). The presence of antibodies alone did not modify cell proliferation (Figure 5). Our results indicated that calcitriol decreased cell proliferation by inducing the synthesis of the proinflammatory cytokines IL-1*β* and TNF-*α*.

### 3.5. The Combination of Calcitriol with TNF-*α* Decreased Cell Proliferation in a Greater Extent than Each Compound Alone

**Figure 6:**
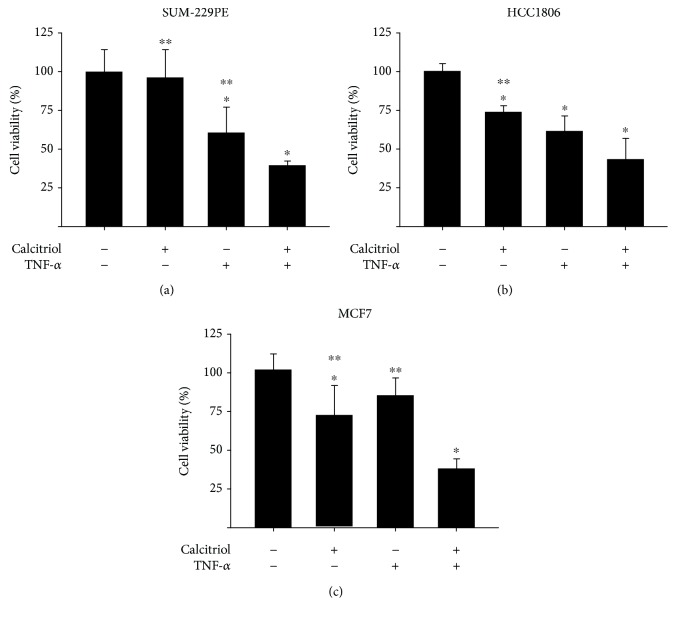
The combination of calcitriol and TNF-*α* inhibited cell proliferation in a greater extent than each compound alone. SUM-229PE (a), HCC1806 (b), and MCF7 (c) cells were incubated with calcitriol, TNF-*α*, or their combination during 6 days, and cell proliferation was evaluated. Calcitriol was used at the concentrations of 0.1, 100, and 10 nM in SUM-229PE, HCC1806, and MCF7 cells, respectively, and TNF-*α* at a concentration of 5 ng/ml in all cases. Data from vehicle-treated cells (-) were normalized to 100%. The results represent the average of 3 experiments, each in triplicate ± SD. ^∗^ *P* < 0.05 vs. vehicle and ^∗∗^ *P* < 0.05 vs. combined treatment.

It has been demonstrated that calcitriol and its analogues improve the antiproliferative response of therapeutic agents and potentiate TNF-*α*-induced cytotoxicity on breast cancer cells [31, 33, 39]. Moreover, considering that both calcitriol and TNF-*α* inhibited cell proliferation in the three established breast cancer cell lines used in this study, we decided to evaluate the combination of both compounds on cell growth. Figure 5 shows the results obtained when calcitriol was combined with TNF-*α*. The simultaneous treatment further inhibited cell growth compared to the compounds alone (Figures 6(a)–6(c)).

Also, the combinatory effect of calcitriol with IL-1*β* in cell proliferation was evaluated; however, there were no significant changes between treatments alone and in combination (data not shown).

## 4. Discussion

TNBC represents a challenge for the development of therapeutic strategies due to the degree of cell dedifferentiation and the dysregulation of molecules involved in the control of proliferation, apoptosis, migration, invasion, and immune response [4]. Vitamin D deficiency has been associated with an increased risk of developing breast cancer [18, 40]. In fact, low levels of calcitriol or its precursor calcidiol have been significantly associated with TNBC in African-American women, including several autoimmune and chronic inflammatory disorders [18, 41, 42]. In addition to its well-known antitumor and antiproliferative functions, calcitriol exerts immunomodulatory effects that result in the prevention of an exacerbated immune response and induction of innate immunity [23]. In regard to IL-1*β* and TNF-*α* regulation by calcitriol, it has been demonstrated that this hormone enhanced muramyl dipeptide-induced TNF-*α* production in monocyte-derived dendritic cells from Crohn's disease patients [43]. Moreover, both calcitriol and its precursor, calcidiol, induced IL-1*β* secretion in monocytic cells [44]. Different effects of calcitriol upon TNF-*α* and IL-1*β* have also been reported in the human placenta [26, 29, 30]. However, the regulation of IL-1*β* and TNF-*α* by calcitriol in TNBC has not been studied. In the present work, we demonstrated for the first time that TNBC cells expressed IL-1*β* and TNF-*α* receptors. These cells also expressed the VDR, as previously shown [45]. Accordingly, our data support that calcitriol and its analogue exert immunomodulatory effects on these cells, being that both increased IL-1*β* and TNF-*α* mRNA and secretion in SUM-229PE cells and IL-1*β* levels in HCC1806 cells. Also, we found that our cultured cells responded to IL-1*β* and TNF-*α* in terms of cell proliferation. In this regard, these two cytokines inhibited cell growth, in a similar manner than calcitriol. However, controversial effects have been attributed to IL-1*β* and TNF-*α* in breast cancer [8, 14, 46, 47]. These controversial results are difficult to explain; however, variances in the cellular type and context, experimental conditions, and culture microenvironment could be taken into consideration to explain the differential responses to IL-1*β* and TNF-*α* on our cultured breast cancer cells. Indeed, these cytokines significantly inhibited or did not change proliferation depending on the concentration and cell line evaluated. Similar to our results, the antiproliferative functions of these cytokines were also demonstrated, in the same concentrations, in MCF7 cells herein and elsewhere [14]. From a clinical perspective, we found that in TCGA data retrieved from the Human Protein Atlas database, breast cancer patients with elevated expression of IL-1*β* had better prognosis reflected in the OS.

Regarding calcitriol and its analogue, and as expected, both compounds decremented SUM-229PE and MCF7 cell proliferation [32, 34]; however, they did not affect the growth of HCC1806 cells. In this study, SUM-229PE cells were more sensitive to calcitriol and cytokines when compared to HCC1806 cells. Indeed, although both lines are TNBC cells, SUM-229PE cells belong to the basal-like 1 (BL1) subtype, whereas HCC1806 cells to basal-like 2 (BL2). It is known that the BL1 subtype is characterized by increased proliferation, loss of cell cycle control, and high expression of genes responding to DNA damage [6], while the BL2 subtype is distinguished by high expression of myoepithelial markers and increased growth factor signaling. In addition, the BL2 subtype does not respond to any classical treatment [48]. Taking these observations into consideration, the cytokines as well as calcitriol and its analogue could be inhibiting proliferation in SUM-229PE cells by inducing cell cycle arrest, as it has been observed in other tissues [49–52]. This requires further investigation.

Since in this study, both IL-*β* and TNF-*α* inhibited cell proliferation and their synthesis was stimulated by calcitriol, we hypothesized that the antiproliferative effects of calcitriol could be partially carried out by regulating endogenous production of IL-1*β* and TNF-*α* in SUM-229PE TNBC cells. Our results demonstrated that when the action of IL-1R1 and TNFR1 was inhibited with specific antibodies, the inhibitory effect of calcitriol was significantly abolished, strongly supporting our hypothesis. Regarding the specific cytokine receptor involved in this effect, the reversibility of the growth inhibitory actions of calcitriol by anti-IL-1R1 and anti-TNFR1 antibodies was expected, given that IL-1*β* signaling is known to require mainly IL-1R1, since IL-1R2 acts rather as a decoy receptor [10, 11], and TNFR1 activation mainly induces apoptosis, in contrast with TNFR2 that promotes cell proliferation [15–17]. In fact, SUM-229PE cells had higher IL-1R1 and TNFR1 protein expression when compared to IL-1R2 and TNFR2, which could be one of the factors contributing to signaling by these receptors. Opposed to this mechanism of action of calcitriol found in our study, Peleg et al. demonstrated that calcitriol and some analogues blocked IL-1*β*-induced growth of acute myelogenous leukemia progenitor cells [27]. The above observations indicate that the cellular context, conditions, and microenvironment play a role in calcitriol and cytokine signaling and their final biological effects. Specifically, our results demonstrated that calcitriol induces IL-1*β* and TNF-*α* production, which, acting in an autocrine fashion through IL-1R1 and TNFR1, inhibits TNBC cell proliferation. Collectively, our data demonstrated an additional mechanism of action by which calcitriol exerts antiproliferative effects, highlighting immunomodulatory and antiproliferative functions of this hormone in the TNBC tumor subtype.

In recent years, combination regimens of drugs have improved treatment outcomes in cancer. In this regard, our laboratory and others have clearly shown the effects of calcitriol on cell proliferation in a variety of cancer cell lines, particularly when combined with other well-established cancer therapies [32–34]. On the other hand, TNF-*α* combined with radiotherapy or cryosurgery results in a synergistic antitumor response or complete tumor destruction, respectively, in breast cancer models [53, 54]. Interestingly, the pretreatment with calcitriol analogues potentiated TNF-*α* cytotoxic effects on ER-positive breast cancer cells in terms of loss of cell viability and DNA fragmentation [31]. In a similar way, in this study, we demonstrated that the combination of calcitriol with TNF-*α* resulted in a greater antiproliferative effect than drug alone in all breast cancer cells evaluated. Notably, HCC1806 cells, which were less sensitive to calcitriol, showed a significant reduction in cell proliferation when exposed to the compound combination. Possibly, the combined treatment of calcitriol and TNF-*α* improved the growth inhibitory response of cells due to the ability of calcitriol to increase TNF-*α*-induced apoptosis, as it had been previously demonstrated with vitamin D derivatives in MCF7 cells by Pirianov and Colston [31].

## 5. Conclusions

The data presented herein indicated that, mechanistically, the antiproliferative actions of calcitriol involve the participation of the endogenous proinflammatory cytokines IL-1*β* and TNF-*α* in TNBC cells. These results are of particular importance, especially for their implications in the treatment of some breast cancers, such as those bearing a triple-negative phenotype.

## Figures and Tables

**Table 1:** Probes and oligonucleotides used in qPCR assays.

Gene	Sense oligonucleotide	Antisense oligonucleotide	Fragment generated (bp)	Probe number
IL-1*β*	TAC CTG TCC TGC GTG TTG AA	TCT TTG GGT AAT TTT TGG GAT CT	76	78
TNF-*α*	CAG CCT CTT CTC CTT CCT GA	GCC AGA GGG CTG ATT AGA GA	123	29
VDR	GTG AGA CCT CAC AGA AGA GCA C	CAT TGC CTC CAT CCC TGA	72	68
GAPDH	AGC CAC ATC GCT CAG ACA C	GCC CAA TAC GAC CAA ATC C	66	60

**Table 2:** IL-*β* and TNF-*α* secretion induced by calcitriol and its analogue in breast cancer cell lines.

		SUM-229PE		HCC1806		MCF7	
		IL-1*β* (pg/ml)	TNF-*α* (pg/ml)	IL-1*β* (pg/ml)	TNF-*α* (pg/ml)	IL-1*β* (pg/ml)	TNF-*α* (pg/ml)
Calcitriol (nM)	0	14.9 ± 8.9	7.1 ± 2.2	29.2 ± 2.5	28.4 ± 9.9	15.4 ± 4.9	27.9 ± 10.5
	1	16.3 ± 1.2	10.5 ± 1.7	24.9 ± 1.7	23.7 ± 0.4	13.8 ± 4.0	34.3 ± 9.9
	10	19.1 ± 2.3	22.9 ± 6.6^∗^	31.3 ± 1.8	19.9 ± 1.7	12.8 ± 2.4	26.7 ± 3.1
	100	33.4 ± 2.4^∗^	10.9 ± 3.1	32.1 ± 3.4	20.3 ± 1.0	20.5 ± 3.4	36.4 ± 8.2

EB1089 (nM)	1	39.2 ± 11.2^∗^	13.1 ± 1.3^∗^	46.4 ± 3.4^∗^	23.5 ± 6.3	11.76 ± 4.5	24.5 ± 8.6
	10	24.3 ± 0.9^∗^	17.9 ± 0.9^∗^	41.7 ± 3.4^∗^	21.3 ± 6.2	13.54 ± 1.3	25.0 ± 11.4
	100	27.4 ± 5.1^∗^	17.7 ± 1.6^∗^	38.7 ± 0.8^∗^	28.4 ± 7.3	17.84 ± 6.6	35.7 ± 0.7

Results are expressed as the mean ± SD cytokine secretion of triplicate determinations and represent at least three different experiments. ^∗^ *P* < 0.05 vs. nontreated cells (0).

## Data Availability

The data used to support the findings of this study are available from the corresponding author upon request.

## Supplementary Materials

Supplementary Materials Supplementary 1. Figure S1: effect of calcitriol and its analogue on IL-1*β* and TNF-*α* gene expression in breast cancer cells. HCC1806 (a, b) and MCF7 (c, d) cells were cultured in the absence or presence of different concentrations of calcitriol (black bars) or EB1089 (white bars) for 24 hours. IL-1*β* (a, c) and TNF-*α* (b, d) gene expression was assessed by qPCR. The results represent the average of at least 3 experiments in triplicate ± SD. ^∗^ *P* < 0.05 vs. control. The value of the cells without treatment was normalized to one for gene expression.
