# Supplementary material for: Calcitriol Inhibits the Proliferation of Triple-Negative Breast Cancer Cells through a Mechanism Involving the Proinflammatory Cytokines IL-1β and TNF-α
Source: J Immunol Res. 2019 Apr 10;2019:6384278. doi: 10.1155/2019/6384278 (PMC6481021; doi:10.1155/2019/6384278)
Supplement: Supplementary Materials — Supplementary 1. Figure S1: effect of calcitriol and its analogue on IL-1β and TNF-α gene expression in breast cancer cells. HCC1806 (a, b) and MCF7 (c, d) cells were cultured in the absence or presence of different concentrations of calcitriol (black bars) or EB1089 (white bars) for 24 hours. IL-1β (a, c) and TNF-α (b, d) gene expression was assessed by qPCR. The results represent the average of at least 3 experiments in triplicate ± SD. ∗ P < 0.05 vs. control. The value of the cells without treatment was normalized to one for gene expression. [file 6384278.f1.pdf]

Supplementary Materials

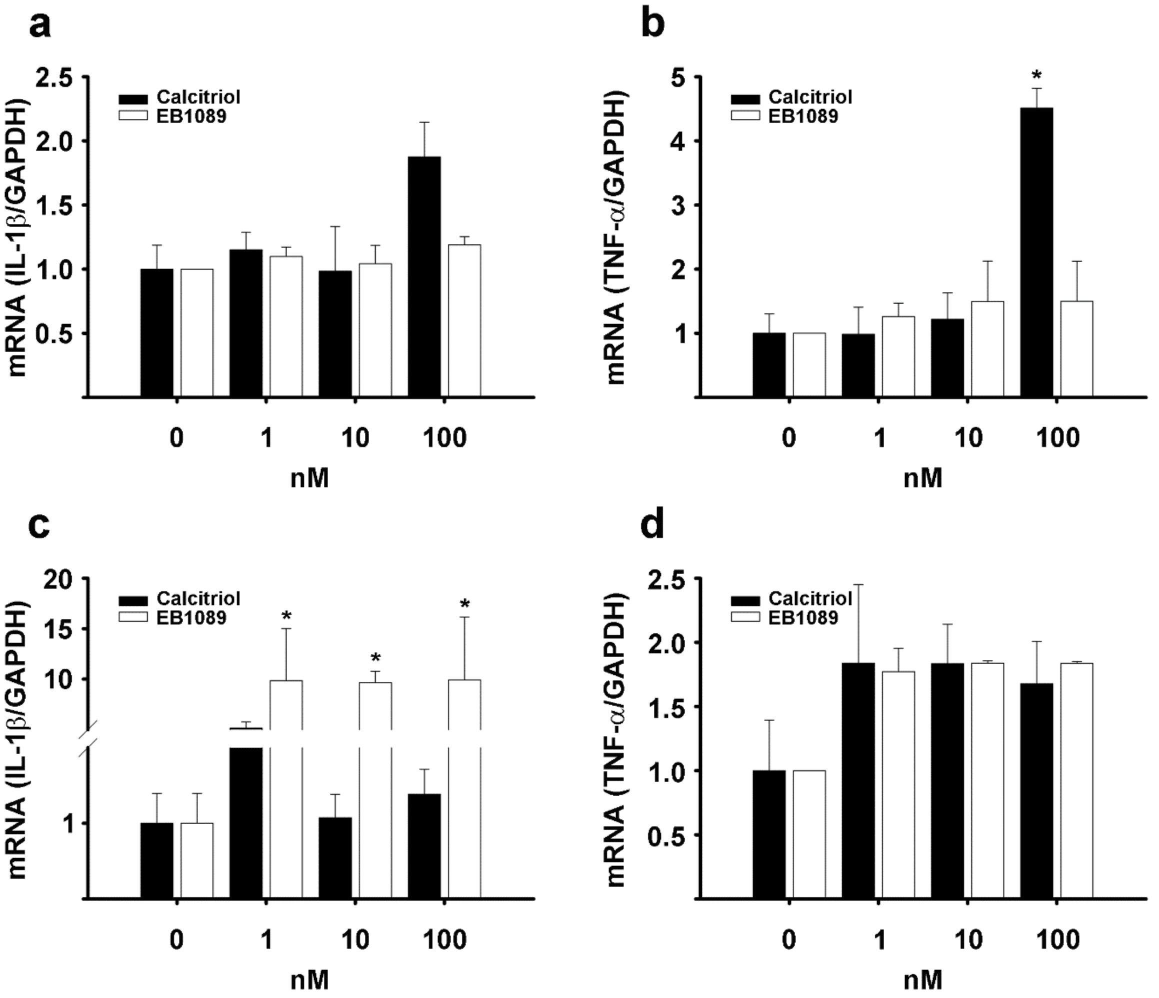

Supplementary 1. Figure S1: Effect of calcitriol and its analogue on IL-1β and TNF-α gene expression in breast cancer cells. HCC1806 (a and b) and MCF7 (c and d) cells were cultured in the absence or presence of different concentrations of calcitriol (black bars) or EB1089 (white bars) for 24 hours. IL-1β (a and c) and TNF-α (b and d) gene expression was assessed by qPCR. The results represent the average of at least 3 experiments in triplicate ± S.D. *P <0.05 vs control. The value of the cells without treatment was normalized to one for gene expression.
